# Supplementary material for: Quantum-disordered state of magnetic and electric dipoles in an organic Mott system
Source: Nat Commun. 2017 Nov 28;8:1821. doi: 10.1038/s41467-017-01849-x (PMC5703743; doi:10.1038/s41467-017-01849-x)
Supplement: Supplementary file 1 — Supplementary Information [file 41467_2017_1849_MOESM1_ESM.pdf]

Supplementary Note 1: Sample, d.c. bias, and frequency dependence of dielectric permitivity

To check the sample dependence of the observed quantum paraelectric (QPE) behaviour in H-Cat, we measured the dielectric permittivity for several samples (the data of crystal #1 is shown in the main text). As shown in Supplementary Fig. 1a, there is no significant sample dependence in $\epsilon_{\rm r}$; the observed small difference is due to the inaccuracy in sample dimensions and is within the experimental error. We also examined the d.c. bias dependence of the dielectric permittivity in H-Cat. In addition to the a.c. electric field, a d.c. electric field of 1 $\rm kV\,cm^{-1}$ for both crystal #2 and crystal #3 was applied. As shown in Supplementary Fig. 1a, the application of d.c. bias electric field has no detectable influence on the QPE behaviour in H-Cat. Moreover, we measured the frequency dependence of the dielectric permittivity in H-Cat (100 Hz to 1 MHz). As shown in Supplementary Fig. 1b, the dielectric permittivity is frequency-independent below $\sim$ 10 K, which supports that the observed QPE behaviour is intrinsic. In contrast, a strong frequency dependence is observed above $\sim$ 10 K, which originates from a large loss tangent due to the relatively high electrical conductivity in H-Cat. Such an extrinsic effect on the dielectric behaviour is often observed in organic conductors with large loss tangents. Because the loss tangent $\tan\delta$ is given by $1/(\omega C R_{\rm p})$, where ω is the angular frequency of the a.c. field, C is the lossless capacitance, and $R_{\rm p}$ is the parasitic resistance, the higher the measuring frequency is, the smaller the dielectric loss becomes. Therefore, we used the dielectric permittivity data measured at 1 MHz to obtain the physical parameters such as T_0 and T_1 in the Barrett formula.

Supplementary Note 2: Resonance scattering arising from hydrogen-bond dynamics

To investigate how the phonon thermal conductivity is suppressed in H-Cat, we applied the Callaway model that describes a phonon thermal conductivity $\kappa_{\rm ph}$ [1]:

$$\kappa_{\rm ph} = \frac{k_{\rm B}}{2\pi^2 v_{\rm ph}}\left(\frac{k_{\rm B}}{\hbar}\right)^3 T^3 \int_0^{\Theta_{\rm D}/T} \frac{x^4 e^x}{(e^2-1)^2}\tau(\omega,T)dx, \quad (1)$$

where $v_{\rm ph}$ is the average sound velocity of the phonons, $\Theta_{\rm D}$ is the Debye temperature, τ is the total phonon relaxation rate and ω is the frequency of phonons; moreover, $\hbar$ and $k_{\rm B}$

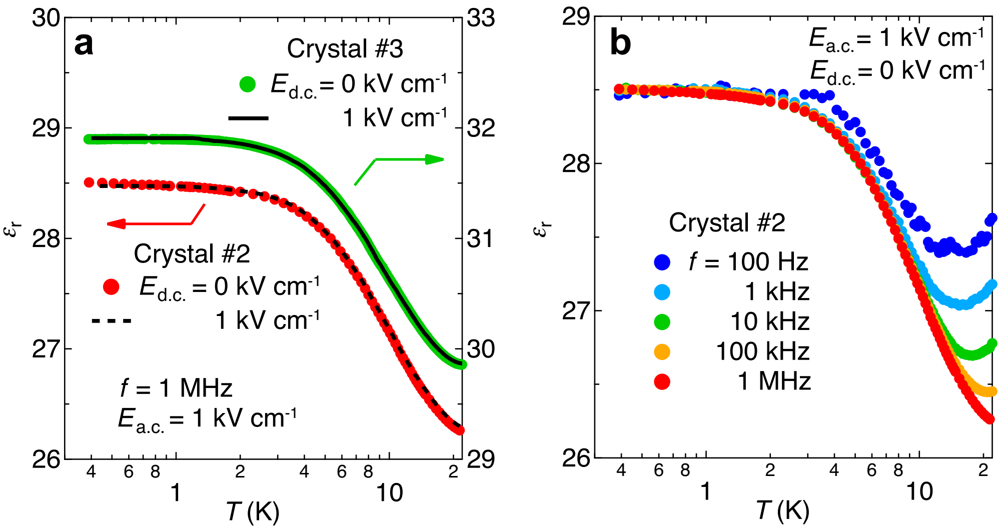

Supplementary Figure 1. a, Temperature dependence of the dielectric constant for two crystals of H-Cat (#2 and #3) measured at an a.c. electric field of 1 kV cm^{-1} with a frequency of 1 MHz. The dielectric constant measured with a d.c. bias electric field of 1 kV cm^{-1} is also plotted. **b,** Frequency dependence of the dielectric constant for crystal #2.

are the reduced Planck and Bolzmann constants, respectively, and $x = \frac{\hbar\omega}{k_B T}$. By fitting the Debye model to the specific-heat data [2], the values of v_{ph} and Θ_D were estimated as 1.7 km s^{-1} and 220 K, respectively. $\tau(\omega, T)$ was approximated as [3, 4]:

$$1/\tau(\omega,T) = \frac{v_{ph}}{L} + A\omega + D\omega^4 + B\omega^2 T \exp\left(-\frac{\Theta_D}{fT}\right) + g\left(\frac{\omega^4}{\omega^2-\omega_0^2}\right)^2\left[1 - h\tanh^2\left(\frac{\hbar\omega_0}{2k_B T}\right)\right], \tag{2}$$

where the first three terms represent the phonon scatterings at the sample boundaries, dislocations and point defects, respectively, and the last two terms stand for the scatterings caused by phonon–phonon (Umklapp) processes and a resonance mode, respectively. Here L, A, D, B, f, g and h are constant coefficients and ω_0 is the resonance frequency. Using this model, we evaluated the phonon thermal conductivity of H-Cat above 2 K, where the spin contribution κ_{sp} is small. For comparison, we also analyzed the thermal conductivity of D-Cat in the same temperature range.

As shown in Supplementary Fig. 2, $\kappa(T)$ for D-Cat is fitted by the above $\tau(\omega, T)$ without a resonance mode. In H-Cat, $\kappa(T)$ is well reproduced only by adding a single resonance scattering process with an energy of $\hbar\omega_0/k_B \sim 8$ K to the fitting result of D-Cat. The fitting parameters (summarized in Supplementary Table 1) are in good agreement with those of

	L (m)	A	D (s^3)	B ($K^{-1}s$)	f	g (s^{-1})	h	$\frac{\hbar\omega_0}{k_B}$ (K)
H-Cat	0.3×10^{-3}	3.64×10^{-4}	8.04×10^{-41}	2.32×10^{-14}	2.65	1.47×10^{9}	0.975	8
D-Cat	0.3×10^{-3}	3.64×10^{-4}	8.04×10^{-41}	2.32×10^{-14}	2.65	–	–	–

Supplementary Table 1. Fitting parameters of Supplementary Eqs (1) and (2) for the thermal conductivity data.

other quantum spin systems [3]. This result indicates that the resonance mode strongly suppresses the phonon thermal conductivity of H-Cat. As H-Cat possesses both proton and spin degrees of freedom, this extra resonance scattering comes from either proton–phonon scattering or spin–phonon scattering. The resonance scattering energy almost coincides with the proton fluctuations ($T_1 = 7.7$ K), which suggests that the resonance scattering arises from the optical mode associated with the hydrogen bonds. Here, we can safely exclude spin–phonon scattering as the origin of the resonance scattering because of the following reasons. The spin–phonon scattering should be strongly quenched by Zeeman splitting under a magnetic field, which changes the field dependence of the thermal conductivity $\kappa(H)$. However, $\kappa(H)$ does not strongly depend on the magnetic field up to 10 T (see Fig. 4a). Moreover, the g-value only slightly deviates from 2 and thereby supports that the spin–orbit coupling causing the spin–phonon scattering is very weak [5]. Thus, we conclude that the strong suppression of κ_{ph} for H-Cat results from resonance scattering, which itself arises from the hydrogen-bond dynamics.

Supplementary Note 3: Specular scattering at sample surfaces

Here, we explain the observed T^2-dependence of κ_{ph} in H-Cat and D-Cat below ~ 0.25 K. According to the kinetic equation, κ_{ph} depends on the heat capacity of the phonons C_{ph}, the sound velocity v_{ph} and the mean free path of phonon l_{ph}:

$$\kappa_{\text{ph}} = \frac{1}{3} C_{\text{ph}} v_{\text{ph}} l_{\text{ph}}. \tag{3}$$

In general, l_{ph} and v_{ph} become essentially constant with temperature and C_{ph} varies as $C_{\text{ph}} \propto T^3$ at temperatures significantly lower than Θ_{D}, leading to the relation $\kappa_{\text{ph}} \propto T^3$. However, we found that $\kappa_{\text{ph}} \propto T^2$ holds true at sufficiently low temperatures in both H-Cat and D-Cat even though the specific-heat measurements supports $C_{\text{ph}} \propto T^3$ [2]. Such unexpected

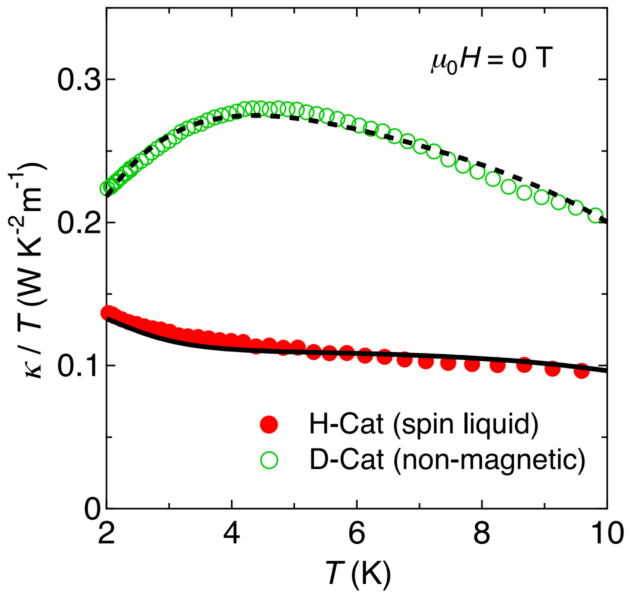

Supplementary Figure 2. Temperature dependence of κ/T for H-Cat (filled red symbols) and D-Cat (open green symbols) over the temperature range 2–10 K in the zero-field. Solid and dashed lines are the Callaway model fits to H-Cat and D-Cat data, respectively.

behaviours are often observed in glassy materials [6] or in high-quality crystals with specular surfaces [7–9]; in these cases, $l_{\rm ph}$ continues to increase with decreasing temperature. In glassy materials, impurity scattering reduces $l_{\rm ph}$ to below the width or thickness of the sample, whereas in high-quality crystals, $l_{\rm ph}$ can exceed the sample size. Here, we estimated $l_{\rm ph}$ of D-Cat, for which $\kappa = \kappa_{\rm ph}$. As shown in Supplementary Fig. 3, $l_{\rm ph}$ of D-Cat continues to increase with decreasing temperature and extends well beyond the sample boundaries assuming that the value of $v_{\rm ph}$ ($\sim$ 1.7 km s^{-1}) remains independent of temperature. This result supports the occurrence of specular surface scattering in the present system, which is the cause of the T^2-dependence of $\kappa_{\rm ph}$.

Supplementary Note 4: Thermal properties of itinerant spin excitations

We investigated the low-lying spin excitation spectrum that characterizes the quantum spin liquid (QSL) states. The spin contribution $\kappa_{\rm sp}$ in H-Cat is extracted by subtracting the phonon contribution $\kappa_{\rm ph}$ from the total thermal conductivity $\kappa = \kappa_{\rm sp} + \kappa_{\rm ph}$. As shown by dashed lines in Fig. 3b, κ/T at low temperatures is well fitted to the formula $\kappa/T = \eta_0 + \beta T$, where η_0 and β are constant coefficients. A finite residual η_0 of $\sim$ 0.06 W K^{-2}m^{-1} is clearly

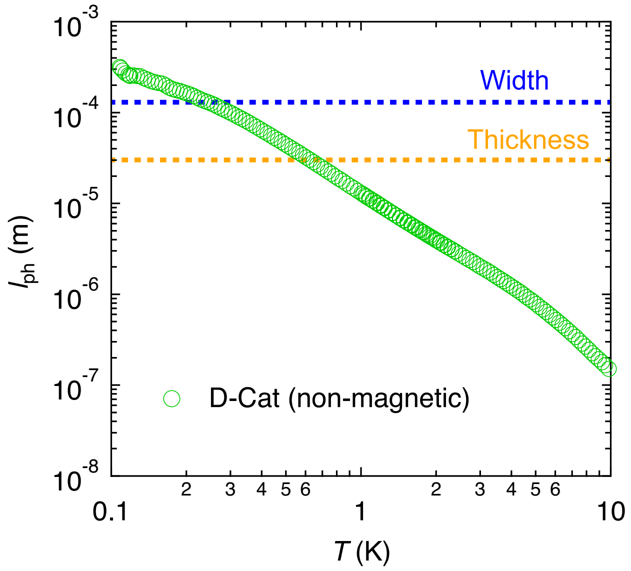

Supplementary Figure 3. Temperature dependence of l_{ph} for D-Cat. Blue and orange dotted lines represent the typical width and thickness of single crystals of D-Cat, respectively.

resolved in the zero-temperature limit (Fig. 3b). We note that the residual term is also finite ($\sim$ 0.11 W K^{-2}m^{-1}; see Supplementary Fig. 4) despite application of the conventional form $\kappa/T = \eta_0 + \beta T^2$. The residual κ/T shows that the spin excitation from the ground state is gapless.

We next estimated the mean free path of the spin excitations l_{sp} [10]. Analogous to the case of phonons, we assumed the following relation:

$$\frac{\kappa_{sp}}{T} = \frac{1}{3}\frac{C_{sp}}{T} v_{sp} l_{sp}, \tag{4}$$

where C_{sp} is the specific heat and v_{sp} is the velocity of the spin excitations. Here, we adopted the standard formula of 2D fermions:

$$\frac{C_{sp}}{T} = \frac{\pi^2}{3} k_B^2 N_F \frac{1}{d}, \tag{5}$$

where N_F is the density of states at the Fermi surface and d = 1.47 Å is the interlayer spacing. We also assumed that the linear term η_0 arises from fermionic excitations. In this case, the Fermi wave number is written as $k_F = 1/a$, where a = 8.36 Å is the nearest-neighbour spin distance. From the above equations, we obtained $\eta_0 = \frac{\pi}{9}\frac{k_B^2}{\hbar}\frac{l_{sp}}{ad}$. Because the estimated residual η_0 is at least 0.06 W K^{-2}m^{-1} (see the inset of Supplementary Fig. 4), we found that l_{sp} exceeds 120 nm at zero temperature; that is, the spin excitations are mobile

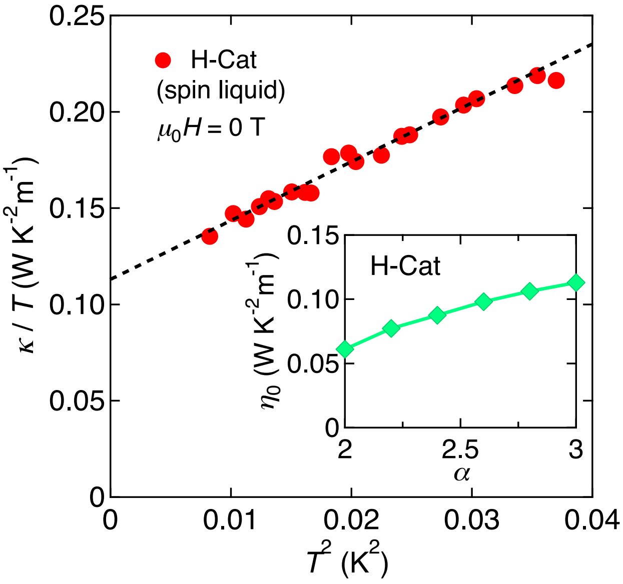

Supplementary Figure 4. Low-temperature plot of κ/T as a function of T^2 in H-Cat. The dashed line represents a fit to $\kappa/T = \eta_0 + \beta T^2$, where η_0 and β are constant coefficients. Inset: η_0 versus the exponent α, where $\kappa/T = \eta_0 + \beta T^{\alpha-1}$. A finite η_0 is clearly observed for all α. The minimum and maximum values of η_0 at $\alpha = 2$–3 are approximately 0.06 and 0.11 $\mathrm{W\,K^{-2}m^{-1}}$, respectively.

to a distance 100 times the inter-spin distance without being scattered. Such highly mobile spin excitations apparently result from the extremely long spin correlation length.

Supplementary Note 5: Origin of itinerant low-energy excitations in thermal conductivity of H-Cat

The observed negligibly small field dependence of the thermal conductivity in H-Cat may raise a question as to the magnetic origin of the itinerant low-lying excitations observed in the thermal conductivity of H-Cat. However, we can exclude the possibility that the itinerant low-energy excitations, which are described by the T-linear term of the thermal conductivity as discussed in the main text, is due to either phonons or electric dipoles for the following reasons: (1) As shown in the dotted lines in Fig. 3b, the temperature dependence of κ/T of both H-Cat and D-Cat exhibits a T-linear dependence at low temperatures. Considering that D-Cat has only phonon contribution to the thermal conductivity, this result indicates that the phonon thermal conductivity in this system is described by a T^2-dependence (owing

to the influence of high-quality crystals with specular surfaces, for details, see Supplementary Note 3) rather than the conventional T^3-dependence. Because $\kappa_{\rm ph}/T \propto T$ goes to zero in the zero-temperature limit, the phonon thermal conductivity cannot contribute to the itinerant low-energy excitation observed in H-Cat. (2) Because electric dipoles behave as classical Ising-like spins, a dielectric system with 1D uniaxial (Ising-type) antiferroelectric dipoles, as in the case of H-Cat, is expected to have low-energy excitations with a gap, which is inconsistent with the gapless excitations in H-Cat.

Supplementary Note 6: Origin of peak structure observed in thermal conductivity of H-CAT

The peak structure observed in $\kappa^{\rm H}/T$ around 0.3 K originates from both $\kappa^{\rm H}_{\rm sp}$ and $\kappa^{\rm H}_{\rm ph}$. We first focus on $\kappa_{\rm sp}$ described by Supplementary Eq. (4). Recent torque measurements [5] suggest that below 2 K, the spin correlation develops in the QSL state; this implies that $l_{\rm sp}$ increases with decreasing temperature below 2 K; however, $l_{\rm sp}$ is eventually saturated and reaches ~120 nm at low temperatures. In addition, $C_{\rm sp}/T$ has been reported to be almost constant with temperature below 2 K [2], showing $C_{\rm sp} \propto T$, and $v_{\rm sp}$ is assumed to be essentially temperature-independent at low temperatures. Therefore, $\kappa^{\rm H}_{\rm sp}/T$ below 2 K increases with decreasing temperature and should be saturated at low temperatures without showing any peak structure, which requires $\kappa^{\rm H}_{\rm ph}$ in addition to $\kappa^{\rm H}_{\rm sp}$ to explain the observed peak structure in $\kappa^{\rm H}/T$. As described in Supplementary Note 3, $\kappa^{\rm H}_{\rm ph}/T$ decreases monotonically with decreasing temperature at least at low temperatures because of $\kappa^{\rm H}_{\rm ph}/T \propto T^{\alpha-1}$ with $\alpha = 2$–3. Consequently, we conclude that a combination of $\kappa^{\rm H}_{\rm sp}$ and $\kappa^{\rm H}_{\rm ph}$ results in the peak structure observed in $\kappa^{\rm H}/T$.

Supplementary Note 7: Estimation of distance from Mott transition

The distance from a Mott transition can be estimated from the ratio of the on-site Coulomb repulsion U to the transfer integral t, which is given by $U/t \sim t/J$, where J is the spin interaction. The value of t is calculated to be 30–40 meV for H-Cat [11], ~ 50 meV for κ-(BEDT-TTF)$_2$Cu$_2$(CN)$_3$ [12, 13] and 25–40 meV for EtMe$_3$Sb[Pd(dmit)$_2$]$_2$ [14]. Note that t is comparable among the three materials. In contrast, $J/k_{\rm B}$ of H-Cat is

estimated as $\sim$ 80 K [5] and as $\sim 1/3$ in the other two compounds ($J/k_{\rm B} \sim 250$ K for both κ-(BEDT-TTF)$_2$Cu$_2$(CN)$_3$ [15] and EtMe$_3$Sb[Pd(dmit)$_2$]$_2$ [16]). Consequently, H-Cat has a relatively large U/t; this indicates a large distance from the Mott transition.

Supplementary Note 8: Theoretical model of electron-proton coupled system

We introduce a minimal model that describes a coupling between the π-electron and proton degrees of freedom in H-Cat and D-Cat. The model Hamiltonian is given by

$$\mathcal{H} = \mathcal{H}_{\rm e} + \mathcal{H}_{\rm p}, \tag{6}$$

where the first term $\mathcal{H}_{\rm e}$ describes the π-electron system and the second term $\mathcal{H}_{\rm p}$ represents the proton system coupled with the π-electrons. The first term is given by the extended Hubbard model (EHM):

$$\begin{aligned}\mathcal{H}_{\rm e} = t_{\rm d}\sum_{i\sigma}(c^\dagger_{i\alpha\sigma}c_{i\beta\sigma} + {\rm H.c.}) + \sum_{\langle ij\rangle\sigma} t^{\mu\mu'}_{ij}(c^\dagger_{i\mu\sigma}c_{j\mu'\sigma} + {\rm H.c.})\\ + U_0\sum_{i\mu} n_{i\mu\uparrow}n_{i\mu\uparrow} + V_{\rm d}\sum_{i} n_{i\alpha}n_{i\beta} + \sum_{\langle ij\rangle} V^{\mu\mu'}_{ij} n_{i\mu}n_{j\mu'},\end{aligned} \tag{7}$$

where $c_{i\mu\sigma}$ is the annihilation operator of a hole with spin $\sigma = (\uparrow,\downarrow)$ at the μ $(= \alpha, \beta)$ molecule of the i-th dimer, $n_{i\mu} \equiv \sum_\sigma n_{i\mu\sigma} \equiv \sum_\sigma c^\dagger_{i\mu\sigma}c_{i\mu\sigma}$ is the number operator and $t_{\rm d}$ ($V_{\rm d}$) is the intra-dimer transfer integral (Coulomb interaction) between the α and β molecules inside a dimer. Moreover, $t^{\mu\mu'}_{ij}$ ($V^{\mu\mu'}_{ij}$) is the inter-dimer transfer integral (Coulomb interaction) between the μ molecule of the i-th dimer and the μ' molecule of the j-th dimer and U_0 is the intra-molecular Coulomb interaction (see Supplementary Fig. 5a). The second term $\mathcal{H}_{\rm p}$ in Supplementary Eq. (6) is described by

$$\mathcal{H}_{\rm p} = 2t_{\rm p}\sum_i P^x_i + \frac{1}{2}g\sum_{\langle ij\rangle}(n_{i\beta} - n_{j\alpha})P^z_i, \tag{8}$$

where $t_{\rm p}$ is the proton tunneling amplitude (proton-fluctuation strength) [17], g (> 0) is the coupling constant between the hole and the hydrogen atom and $\boldsymbol{P}_i$ is a pseudo-spin operator with an amplitude of 1/2 describing the proton degree of freedom at the i-th hydrogen bond. Here, we define the eigenstates of P^z as $|+\rangle$ and $|-\rangle$, respectively. In these states, the hydrogen atom is localized at the right and left sides of the double potential minima, respectively (see Supplementary Fig. 5b). The eigenstates of P^x, denoted by $|A\rangle = (|+\rangle + |-\rangle)/\sqrt{2}$ and

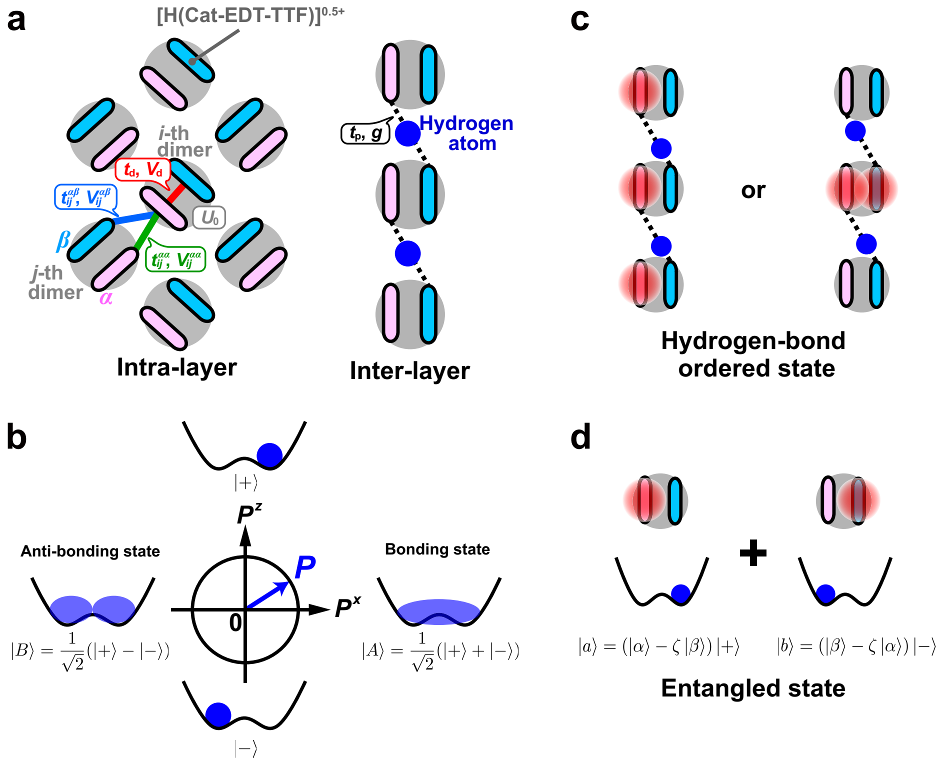

Supplementary Figure 5. **a,** Schematic lattice structures of an intra-layer (left) and inter-layer (right) for H-Cat. The ellipses represent the $[H(Cat\text{-}EDT\text{-}TTF)]^{0.5+}$ molecules and the two molecules in a dimer (gray circle) are labelled α and β. The red, green and blue lines represent the transfer integrals ($t_{\mathrm{d}}, t_{ij}^{\alpha\alpha}$ and $t_{ij}^{\alpha\beta}$) and the Coulomb interactions ($V_{\mathrm{d}}, V_{ij}^{\alpha\alpha}$ and $V_{ij}^{\alpha\beta}$) introduced in the EHM. The black dotted lines indicate proton tunneling and the coupling constant between the hole and the hydrogen atom. **b,** Pseudo-spin directions in the P^x–P^z plane and the corresponding states of the hydrogen atom. **c, d,** Schematic views of hydrogen-bond ordered states (**c**) and an entangled state (**d**) expected in the present theoretical model. For simplicity, we consider the case of $\zeta = 0$. The size of red circles represents a relative hole density.

$|B\rangle = (|+\rangle - |-\rangle)/\sqrt{2}$, represent the bonding and anti-bonding states, respectively (see Supplementary Fig. 5b). This pseudo-spin representation has been applied to hydrogen-bonded systems such as the ferroelectric KH_2PO_4 (KDP) [18, 19], where displacive deformation of PO_4 tetrahedrons is coupled with O-H-O hydrogen dynamics.

We now consider a 1D chain of π-dimers and hydrogen bonds in the strong electron correlation regime, where the on-site Coulomb interaction is larger than the inter-dimer Coulomb interaction. In this situation, for relatively small t_{p}, hydrogen-bond order occurs

concomitantly with charge order inside of the π-dimer, leading to two types of ordering patterns. In one case, the hydrogen atoms are located at the same oxygen side for two O-H-O hydrogen bonds and each hole is distributed to the same molecular side of each dimer (see the left panel of Supplementary Fig. 5c). In the other case, each hydrogen atom is located at the opposite oxygen side, leading to a state where one dimer is occupied by two holes and the other is empty (see the right panel of Supplementary Fig. 5c); this state corresponds to the non-magnetic D-Cat.

In contrast, for relatively large t_p, any hydrogen order does not occur owing to the strong proton tunneling effect, which corresponds to the state for H-Cat. To investigate this state more closely, we consider the simplest case, in which an isolated dimer occupied by one hole is coupled to a proton. In this case, the ground-state wave function is given by an entangled state (see Supplementary Fig. 5d) $\Phi_{1,2} = |a\rangle + |b\rangle = (|\alpha\rangle - \zeta\,|\beta\rangle)\,|+\rangle + (|\beta\rangle - \zeta\,|\alpha\rangle)\,|-\rangle$. Here, $|\alpha\rangle$ and $|\beta\rangle$ represent the states where the π electron is localized at the α and β molecules inside the dimer, respectively. In addition, $|\zeta|$ (< 1) expresses the degree of hybridization of $|\alpha\rangle$ and $|\beta\rangle$ induced by t_d. We stress that when $t_\mathrm{p} \neq 0$, Φ cannot be described by a direct product $\Phi_\mathrm{h} \otimes \Phi_\mathrm{p}$, where $\Phi_{\mathrm{h(p)}}$ is the hole (proton) wavefunction expressed as a linear combination of $|\alpha\rangle$ and $|\beta\rangle$ ($|+\rangle$ and $|-\rangle$), whereas at $t_\mathrm{p} = 0$, the eigenstates of $|a\rangle$ and $|b\rangle$ are degenerate. Such a proton–electron entanglement in the presence of t_p has been confirmed in numerical calculations of the exact diagonalization method in cluster systems. Thus, proton tunneling t_p is an important factor for determining the ground states in the present system.

In the present theoretical model, we have focused not on the direct proton-proton interaction arising from electrostatic and elastic couplings between two neighbouring protons, but on the proton-proton interaction through the electron-proton coupling. One of the main reasons is that the experimental results suggest that the hydrogen bonds in the present system strongly couple with the charge and spin degrees of freedom of π-electrons. In addition, there is no direct chemical bond connecting the two neighbouring protons. This structural feature causes a screening effect of the electrostatic interaction by the conducting π-electrons, as well as a weak elastic interaction between two neighbouring protons. By taking into account these aspects, we adopted the proton-electron interaction as a main interaction responsible for the observed strong correlation between the electron and proton degrees of freedom. To clarify the origin of the QSL state and the effects of the underlying

proton–electron entanglement, further theoretical studies are required.

Supplementary Note 9: Possible explanation for field-independent itinerant spin excitations

To explain negligibly small field dependence of $\kappa_{\rm sp}^{\rm H}$, we consider a gapless spinon Fermi surface over the whole **k**-space. According to the recent torque measurements [5], spin excitations act as Pauli-paramagnetic-like low-energy excitations up to 17 T, which is a hallmark of itinerant fermions (for example, fermionic spinons) at low temperatures. The Pauli susceptibility is given by $\chi = (1/4)g^2\mu_{\rm B}^2 N_{\rm F}$, where $N_{\rm F}$ is the density of states at the Fermi energy $\varepsilon_{\rm F}$, $\mu_{\rm B}$ is the Bohr magneton and g is the g-factor of Cat-EDT-TTF dimer with spin-1/2. Here, we adopt the standard formula in a 2D system $N_{\rm F} = n/\varepsilon_{\rm F}$ [20], where n is the number of fermions (spinons) per volume. Using $g \sim 2$ and $\chi \sim 1.2 \times 10^{-3}$ emu mol^{-1} obtained from the torque measurements for H-Cat [5], the Fermi temperature $T_{\rm F} = \varepsilon_{\rm F}/k_{\rm B}$ is roughly estimated to be $\sim$ 350 K. In such a Pauli-paramagnetic state with a large $T_{\rm F}$ compared to the energy scale of magnetic field, $v_{\rm sp}$ becomes almost field-independent. Meanwhile, in the presence of magnetic field, the spin-up (spin-down) band shifts upward (downward) and the number of spin excitations with up-spin (down-spin) increases (decreases). However, the total number of spin excitations in the applied magnetic field becomes constant in a 2D system owing to the cancellation of the spin-up and spin-down parts, resulting in that $C_{\rm sp}$ is field-independent because of Supplementary Eq. (5), as confirmed by recent specific heat measurements [2]. As a result, if we simply assume that $l_{\rm sp}$ is field-independent, $\kappa_{\rm sp} \propto C_{\rm sp}v_{\rm sp}l_{\rm sp}$ is essential constant with magnetic field.

Supplementary References

[1] Berman, R. *Thermal Conduction in Solids* (Oxford Univ. Press, Oxford, 1976).

[2] Yamashita, S., Nakazawa, Y., Ueda, A. & Mori, H. Thermodynamics of the quantum spin liquid state of the single-component dimer Mott system κ-H_3(Cat-EDT-TTF)$_2$. *Phys. Rev. B* **95**, 184425 (2017).

[3] Sologubenko, A. V., Giannó, K., Ott, H. R., Vietkine, A. & Revcolevschi, A. Heat transport by lattice and spin excitations in the spin-chain compounds $SrCuO_2$ and Sr_2CuO_3. *Phys. Rev. B* **64**, 054412 (2001).

[4] Gofryk, K. *et al.* Anisotropic thermal conductivity in uranium dioxide. *Nature Commun.* **5**, 4551 (2014).

[5] Isono, T. *et al.* Gapless quantum spin liquid in an organic spin-1/2 triangular-lattice κ-H_3(Cat-EDT-TTF)$_2$. *Phys. Rev. Lett.* **112**, 177201 (2014).

[6] Zeller, R. C. & Pohl, R. O. Thermal conductivity and specific heat of noncrystalline solids. *Phys. Rev. B* **4**, 2029–2041 (1971).

[7] Hurst, W. S. & Frankl, D. R. Thermal conductivity of silicon in the boundary scattering regime. *Phys. Rev.* **186**, 801–810 (1969).

[8] Pohl, R. O. & Stritzker, B. Phonon scattering at crystal surfaces. *Phys. Rev. B* **25**, 3608–3614 (1982).

[9] Sutherlamd, M. *et al.* Thermal conductivity across the phase diagram of cuprates: Low-energy quasiparticles and doping dependence of the superconducting gap. *Phys. Rev. B* **67**, 174520 (2003).

[10] Yamashita, M. *et al.* Highly mobile gapless excitations in a two-dimensional candidate quantum spin liquid. *Science* **328**, 1246–1248 (2010).

[11] Tsumuraya, T., Seo, H., Kato, R. & Miyazaki, T. First-principles study of hydrogen-bonded molecular conductor κ-H_3(Cat-EDT-TTF)$_2$. *Phys. Rev. B* **92**, 035102 (2015).

[12] Kandpal, H., Opahle, I., Zhang, Y. Z., Jeschke, H. O. & Valenti, R. Revision of model parameters for κ-type charge transfer salts: An ab initio study. *Phys. Rev. Lett.* **103**, 067004 (2009).

[13] Nakamura, K., Yoshimoto, Y., Kosugi, T., Arita, R. & Imada, M. Ab initio derivation of low-energy model for κ-ET type organic conductors. *J. Phys. Soc. Jpn* **78**, 083710 (2009).

[14] Tsumuraya, T., Seo, H., Tsuchiizu, M., Kato, R. & Miyazaki, T. Cation dependence of the electronic states in molecular triangular lattice system β'-X[Pd(dmit)$_2$]$_2$: A first-principles study. *J. Phys. Soc. Jpn* **82**, 033709 (2013).

[15] Shimizu, Y., Miyagawa, K., Kanoda, K., Maesato, M. & Saito, G. Spin liquid state in an organic Mott insulator with a triangular lattice. *Phys. Rev. Lett.* **91**, 107001 (2003).

[16] Itou, T., Oyamada, A., Maegawa, S., Tamura, M. & Kato, R. Quantum spin liquid in the spin-1/2 triangular antiferromagnet $EtMe_3Sb[Pd(dmit)_2]_2$. *Phys. Rev. B* **77**, 104413 (2008).

[17] Blinc, R. On the isotopic effects in the ferroelectric behaviour of crystals with short hydrogen bonds. *J. Phys. Chem. Solids* **13**, 204–211 (1960).

[18] Vaks, V. G. & Larkin, A. I. On phase transitions of second order. *J. Exptl. Theoret. Phys. (USSR)* **22**, 678–687 (1966).

[19] Larkin, A. I. & Pikin, S. A. On phase transitions of the first order resembling those of the second order. *J. Exptl. Theoret. Phys. (USSR)* **29**, 891–896 (1969).

[20] Watanabe, D. *et al.* Novel Pauli-paramagnetic quantum phase in a Mott insulator. *Nat. Commun.* **3**, 1090 (2012).
